# Therapeutic efficacy of chloroquine for treatment of *Plasmodium vivax *malaria cases in Halaba district, South Ethiopia

**DOI:** 10.1186/1756-3305-4-46

**Published:** 2011-03-31

**Authors:** Tsige Ketema, Kefelegn Getahun, Ketema Bacha

**Affiliations:** 1 Jimma University, College of Natural Sciences, Department of Biology, P. O. Box 378, Jimma, Ethiopia; 2 Jimma University, College of Social Sciences and Law, Department of Geography and Environmental studies, P. O. Box 378, Jimma, Ethiopia

## Abstract

**Background:**

Chloroquine is an anti-malarial drug being used to treat *Plasmodium vivax *malaria cases in Ethiopia. However, emergence of chloroquine resistant strains of the parasite has challenged the current efficacy of the drug. Therefore, the aim of this study was to assess the effectiveness of chloroquine against *P. vivax *strains in one of the malaria endemic areas of Ethiopia, namely Halaba district, located in South Nations and Nationalities Peoples Region (SNNPR) of South Ethiopia

**Results:**

Among 87 malaria patients enrolled in the study, only 80 of them completed the 28-days follow-up. Seven of them dropped from the study for different reasons. Among those study participants that completed their follow-up, 69 were classified under the category of adequate clinical and parasitological response (ACPR). However, the remaining 11 cases were considered as under treatment failure mainly due to recurrence of parasitemia on day 7 (four patients), day 14 (six patients), and day 21 (one patient). The age of all cases of treatment failures was found to be less than 20 years. The load of parasitemia of patients with treatment failure on day of admission (4709.4/μl) was higher than day of recurrence (372.37/μl). Parasite reduction ratio (PRR) of treatment failure cases was 12.6/μl.

**Conclusion:**

This report revealed the rise in treatment failure (13% [95% CI = 0.074 - 0.217]) as compared to earlier reports from Ethiopia. It signals the spreading of chloroquine resistant *P. vivax *(CRPv) strains to malaria endemic areas of Ethiopia. It is recommended that all concerned bodies should act aggressively before further expansion of the current drug resistant malaria.

## Introduction

The efficacies of anti-malarial drugs have been challenged by the emergence of drug resistant strains of *Plasmodium *parasites. For more than 50 years, chloroquine (CQ) was the drug of choice for effective treatment of uncomplicated malaria of all species [1]. Resistance to *Plasmodium falciparium *was first documented in 1986 from the border areas of Ethiopia [2] several years after the first report from Southeast Asia and South America in the late 1950 s [3], followed by gradual spread of the resistant strain throughout the country [4-7]. The high level of CQ resistance of the parasite necessitated change of choice from CQ to sulphadoxine pyramethamine (SP) in 1998. Faster drop in therapeutic efficacy of SP for the treatment of uncomplicated falciparum malaria enforced the adoption of Artemether-lumefanthrine (Coartem^®^) (AL) in place of SP as a first line treatment in 2004 [8,9].

Even though, significant numbers of clinical resistance have been reported for a long time in most parts of Asia, mainly from Indonesia [10-12], chloroquine resistant strains of *P. vivax *was not known in Ethiopia until recent years. It has been used as the first line drug for many years for treatment of *P. vivax *infection. However, after the first report from Debreziet, Ethiopia [13], gradually a few reports are coming from some malaria endemic areas of Ethiopia including Debrezeit (5%) [14], Serbo, 3.6% [15], and Nazreth and Debrezeit (5.76%) [16]. The present study was conducted in the southern part of the country, in Halaba district. Unlike other parts of Ethiopia, the proportions of the two principal etiological agents of malaria (*P. falciparium *and *P. vivax*) in the study area are 30 and 70%, respectively [17]. In addition, there was a report of antimalarial drug resistant strain of *P. falciparium *from the same district [9]. The high prevalence of *P. vivax *malaria and the presence of antimalarial drug resistant *P. falciparium *in the study area could be an indicator for the possible emergence of CRPv strains. In agreement with the hypothesis, the outcome of the study showed the highest ever reported decline in the efficacy of chloroquine drug against *P. vivax*. Except for these few reports, however, the nationwide prevalence of chloroquine resistant *P. vivax *remains poorly studied. This necessitates country wide survey for the current status of chloroquine resistant *P. vivax *malaria in other malaria endemic areas of the country.

## Materials and methods

### Study Site and Period

**Figure 1:**
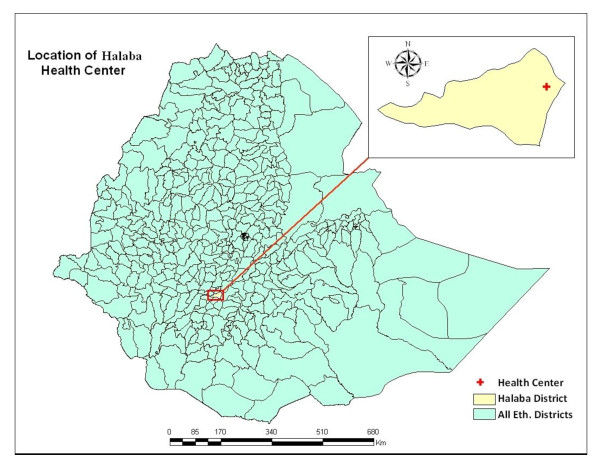
**Map of the study site**.

The clinical trial was conducted at Halaba Kulito Health Center, located in Halaba town about 313 km south of Addis Ababa, the capital city of Ethiopia (Figure 1). The study site is situated within an altitude of ranging from 1554 to 2149 m above sea level, longitude of 38° 7' 0" E and 7° 18' 0" N latitude. The climatic zone of Halaba district consists of mainly mid-land ('*Weinadega*') and low-land ('*Kola*'), which accounts for 86% and 14%, respectively (as reported by the District Agriculture and Rural Development Office). The annual rainfall is estimated to be 857-1085 mm, while the mean annual temperature varies from 17 to 20°C with a mean value of 18°C. The most common health problem in the district is malaria [18]. *Anopheles arabiensis *is a chief vector of the malaria parasite in the region [19]. The study was conducted from January to February, 2009.

### Study Subjects

Individuals seeking treatment for malaria infection at Halaba Kulito Health Center during the study period and having *P. vivax *mono-infection were enrolled in the study. Based on the criteria set by WHO [20], 87 patients who fulfilled the inclusion criteria were recruited for the study following their consent. Since there was no earlier report on CRPv from the study area, the sample size was calculated by assuming a maximum of 5% treatment failures in the population, 5% precision, 95% confidence interval, using the formula, N = (Z/d)^2 ^P (1-P) [21] and 20% expected loss to follow-up over 28 days.

### Inclusion Criteria

The criteria used for inclusion of the study participants were; age >6 months, positive for *P. vivax *mono-infection with parasite density above 250/μl, history of fever during 48 hours prior to time of recruitment, ability and willingness to participate in the study based on information given to parent or guardian, access to health facility and informed consent [20].

### Exclusion Criteria

Presence of clinical conditions that requires hospitalization, presence of severe malnutrition, pregnancy, significant concomitant febrile illness which would interfere with follow-up, chronic infectious diseases other than malaria, known allergy and/or intolerance to drug (s) being tested were the criteria used for exclusion of the patients [20].

### Patient Enrollment, Treatment and Follow-up

Patients confirmed to fulfill the inclusion criteria, and volunteer to participate in the study were treated with chloroquine under supervision of investigators and health professionals working in the health center. Each patient was treated with 25 mg base/kg of chloroquine for three consecutive days [20] followed by seven follow-up days (Day1, 2, 3, 7, 14, 21, and 28). For every follow-up day and on any incident of malaria-like illness, there was an examination of blood film (except on day 1), recording of temperature, and assessment of any health problem and adverse drug reactions. During the follow-up period, any patient who failed to come to the health center on the scheduled date was traced to his/her home assisted by the local guide recruited during the study, and retained in the system unless it was beyond the capacity to trace the lost patient for several reasons (change of address, reluctance to continue as the study subject, etc). In the *in-vivo *follow-up days, patients who failed to respond to chloroquine of therapeutic dose were re-treated with quinine (10 mg base/Kg), the second line drug in Ethiopia.

### Laboratory analysis

#### Drug quality

The status of chloroquine phosphate (Batch number 1482, Adigrat Pharmaceutical Factory, Ethiopia), used to treat all patients was checked for its quality before administered to patients. Accordingly, five tests, including weight variation, disintegration, dissolution, identification and chloroquine hydrochloride injection assay were made, following the standard procedures recommended by British Pharmacopoeia, [22,23], using standard facilities at Drug Administration and Control Authority of Ethiopia (DACA). The drug was confirmed to fulfill all international specifications set for the mentioned tests.

#### Parasite detection

Parasites were identified by microscopic observation of the parasite's morphology using duplicate thick and thin blood smears taken on day of enrolment (Day 0). In addition, blood slides were done at subsequent visits (scheduled and non-scheduled). After fixing the thin film in methanol, both the thin and thick smears were stained with Giemsa (10%, pH~ 7.2) for 30 min and the thick films were examined under oil immersion objective for malaria parasites. *P. vivax *asexual stages were counted against 200 white blood cells (WBC), assuming the median total WBC count of 8,000/μl.

Parasite reduction ratio (PRR) on day of admission and day 2 (after 48 hours) was evaluated as suggested by White [24] using the formula τ = P_o_/P_2 _(where P_o _is parasite count on day 0 and P_2 _parasite count on day 2).

### Quality Assurance

Each slide was carefully examined by experienced laboratory technicians of Halaba Health Center. All *P. vivax *positive and negative slides were re-examined by senior laboratory technician at Jimma University.

### Evaluation of Treatment Responses

The study population were classified into three categories; early treatment failure (in the case of persistence of parasitemia up to day7), late treatment failure (in the presence of parasite recurrence within 28 days follow-up), and non-treatment failure (the absence of parasitemia in 28-days follow-up) [25].

### Data Analysis

Data collected from *in-vivo *therapeutic efficacy test was double entered and analyzed using SPSS software (version 16.0, Chicago, IL, USA). Kaplan-Meier survival probability analysis was used to evaluate treatment outcome of the study participants during follow-up period. Data of patient having mixed infection with *P. falciparum *and vomiting was excluded from the analysis. While data of those patients lost to follow-up were included in the analysis considering them as non-treatment failure cases. In non-normally distributed data (age), median was used to measure the central tendency. Parasite count and parasite reduction ratio were analyzed using geometric mean. In all analysis, significance level was considered at 95% confidence interval.

### Ethical clearance

The study protocol was reviewed and approved by Ethical Review Committee of Jimma University, College of Natural Sciences. Written informed consent was obtained from each patient or their guardians for patient younger than 18 years.

## Results

### Enrollment and Baseline Characteristics

**Figure 2:**
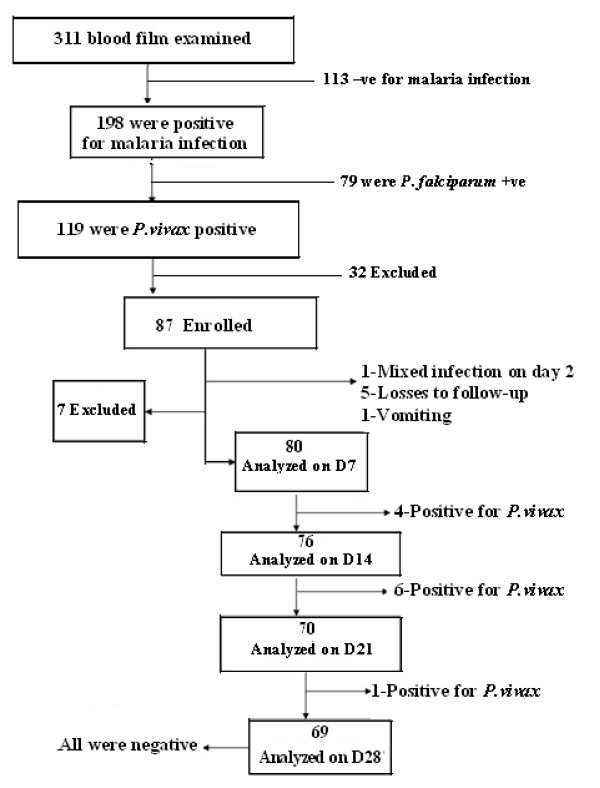
**Flow chart of the study procedure**.

During the enrollment period from January 4 to 10, 2009 (six consecutive days), a total of 311 blood films of patients suspected to have malaria infection were inspected at Halaba Kulito Health Center. A total of 198 cases were positive for malaria infection and 119 cases were confirmed to have *P. vivax *mono-infection. Only 87 of the 119, who fulfilled inclusion criteria set by WHO [20], were included in the study and admitted to the 28 days follow-up (Figure 2). The remaining 32 cases were not enrolled in the study mainly due to lack of willingness, pregnancy, distance of their home from the health center, history of using antimalarial drug prior to seeking medication in the health center, and chronic infection such as TB (Figure 2).

The socio-demographic and some clinical features of the study subjects are as shown below (Table 1). Majority of them were females (57.4%) and their median age was 8 years (9 month - 52 years). The duration of their illness (mean ± SD) before enrollment was 2.84 ± 2.5 days. About 61% had history of fever while 18% had documented fever (axillary temperature ≥37.5°C) on the day of admission. Their geometric mean parasite density was 3072.77/μl.

**Table 1:** Characteristics of *P. vivax *malaria patients enrolled in the *in-vivo *efficacy test of chloroquine in Halaba Kulito Health Center, South Ethiopia, January 2009

Characteristics	Day of admission
Median age in years (proportion)	8 years (9 month - 52 years)
< 5 years (40%)	3 years (9 month - 4 6/12 year)
5-14 years (15.3%)	7 years (5 year - 13 year)
15+ years (44.7%)	26 years (17 year - 52 year)

Sex	
M	42.6% (36/87)
F	57.4% (51/87)

Mean Body Temp (°C).	36.7

History of fever	61% (n = 52)

Axillary Temp. ≥ 37.5°C	18% (n = 15)

Vomiting	11% (n = 9)

Diarrhea	19% (n = 16)

Geometric mean parasite	3072.77/μl

Day of illness (mean ± SD)	2.84 ± 2.5 days

Among 87 patients enrolled in the study, only 69 had completed a 28-days follow-up. Seven of them were excluded from the study at early stage of the follow-up due to loss to follow-up (5 patients), vomiting (1 patient), and mixed infection with *P. falciparium *(1 patient) (Table 2). A total of eleven patients didn't to respond to the therapeutic dose of chloroquine, and they were categorized under treatment failure. As a result, they were re-treated with quinine.

**Table 2:** Response of study participants to chloroquine drug during 28 days follow-up in Halaba Kulite Health Center, South Ethiopia, January 2009.

Follow-up period	At Risk	Excluded from the study for different reasons	Treatment failure	Survival Probability Estimate	0.95 Confidence Interval	
					Lower Limit	Upper Limit
					
Day 0	87	0	0	1	0.9473	1
Day 1	87	4	0	1	0.9473	1

Day 2	84	0	0	1	0.9473	1

Day 3	83	2	0	1	0.9473	1

Day 7	81	0	4	0.9506	0.8755	0.9833

Day 14	72	0	6	0.8714	0.7784	0.9305

Day 21	60	1	1	0.8569	0.7617	0.9197

Day 28	58	0	0	0.8569	0.7616	0.9197

### Treatment Response

Since CQ is a fast acting antimalarial drug, parasitaemia clearance times for almost all patients involved in the 28 days in-vivo study were 48 hours except four patients whose parasitaemia persisted for seven days. Relative PRR of the study participants was 10.7/μl. Similarly, gametocyte was detected for a week in 15 patients and completely cleared on day 14. The study participants were relatively not febrile on day of admission (36.7°C). However, the body temperature dropped with follow-up days. Likewise, patients with treatment failure on day of recurrence were not febrile except three cases (age; 2, 4 and 20). Vomiting and diarrhea, the common malaria symptoms were encountered in 11% and 19% of the cases on day of admission, respectively, and disappeared on day7.

### Characteristics of Treatment Failures

In this study, about 13% (11/85) treatment failure was observed (Table 2). Among the 11 treatment failures, four were males and seven were females. In four of the patients, parasitaemia persisted up to day 7, though the load of parasitaemia was lower than the day of admission. From the early treatment failures, two of the cases (a 20 years old female and a child aged 2 years) had symptom of malaria on the day of recurrence. The other nine treatment failures were observed on day 7 (two), 14 (six) and 21 (one) without showing symptom of malaria. Parasite reduction ratio (PRR) of treatment failure cases was 12.6/μl (Table 3).

**Table 3:** Demographic, Parasitological and Clinical features of patients with treatment failures in Halaba Kulito Health Center, South Ethiopia, January 2009

Characteristics	Proportion
Number of treatment failures	11

Male: Female	4:7

Median Age	7 years

Mean Weight	27.4 Kg

Mean body Temperature	37.6°C

Geometric mean parasite	
On Day 0	4709.4/μl
On Day recurrence	372.37/μl

Parasite Reduction Ratio	12.6/μl

History of Fever	36.36 (n = 4)

Headache	27.2 (n = 3)

Vomiting	54.5 (n = 6)

Diarrhea	45.5 (n = 5)

## Discussion

Chloroquine has been in use both for treatment and prophylaxis in health centers and the communities of Ethiopia. It is an anti-malaria drug recommended by the Ministry of Health of Ethiopia for treatment of *P. vivax *malaria infection in the country. However, some reports on chloroquine resistant *P. vivax *malaria (CRPv) are coming from different malaria endemic areas of Ethiopia [13-16]. In support of the earlier reports, our current study also revealed significantly high treatment failure. Furthermore, some of the other study participants (29%) had complains of malaria symptoms such as headache, fever, chilling, joint pain, and other symptoms during the follow-up period although they were not confirmed by recurrence of parasitemia in their blood. In agreement with earlier reports from Indonesia, Peru, and Ethiopia, most of the treatment failures recorded in this study were children [14,26,27].

As stated by Baird [25], a subject that has an asexual parasitemia of any level persisting to day 7 could be classified as an early treatment failure. According to this definition, four of the treatment failures documented in this study were categorized under early treatment failure and are definitively classified with a demonstration of adequately absorbed, fully compliant therapy. On the other hand, Baird [25] stated that an asexual parasitemia that becomes undetectable but reappears at any time between days 7 and 28 are classified as a recurrence treatment failure. Similarly, seven of the treatment failures recorded in the study on day 14 (six) and 21 (one), should be classified under this category (recurrence treatment failure). Furthermore, Baird [25] revealed that subjects having no recurrent parasitemia up to day 28 should be classified as having an infection that was sensitive to chloroquine. Therefore, 69 of the study participants were possibly infected with chloroquine sensitive strain of *P. vivax*

Treatment failure of *P. vivax *malaria after anti-malarial drug treatment might not necessarily be an indication of the emergence of CRPv strains. There could be other factors contributing to the failure such as malabsorpition of the drug, relapse, poor drug quality, re-infection, low level of drug in blood, and recrudence of parasitemia [28]. In this study, however, the possible causes of the treatment failure were either malabsorption of the drug or recrudescence of parasitemia. Because most of the treatment failures detected in this study were at the early stage of the follow-up (Day 7, and 14, except one on day 21), on these days the blood level of CQ and its metabolites do not drop to level below minimum effective concentration. Thus, it can prevent the recurrence of parasitemia of relapse or re-infection up to 35 days [29].

Besides, for most of the early treatment failures (four), the original parasite persisted up to day 7 though their numbers were less than the day of admission. Under such a situation, it is impossible to think of the treatment failures to be due to re-infection or relapse as a new parasite needs a longer incubation period (ranges from ten to seventeen days). In addition, Baird and Hoffman [30] stated that in tropical areas like Ethiopia, the challenge of a relapse could appear frequently but does not commence before day17. In line with this assumption, relapse could not be the cause for treatment failure documented in this study as 91% of the treatment failures were detected before day 17.

Treatment failures associated with drug resistance may also be due to poor drug quality [31]. However, the quality of drug used to treat all the patients was confirmed in the laboratory using international specifications set for each test. The drug used was confirmed to have good quality and the treatment failures could not be due to poor drug quality.

*In-vivo *therapeutic efficacy studies remain the 'gold standard' method for assessing antimalarial drug efficacy. However, due to the difficulty in classifying true treatment failure, as recurrences of parasitemia (biological resistant) or due to malabsorption of drug by patients, supplementing it with other confirmatory tests is important. For instance measurement of CQ and its major metabolite DCQ in blood of patients with treatment failures could clearly reveal the presence of malabsorption or recurrences of resistant parasites.

## Conclusion

The treatment failure reported in this study (13%) was relatively the highest as compared to earlier few reports from Ethiopia and Africa. This report is a good indicator of the emergence and spread of chloroquine resistant *P. vivax *strains in malaria endemic area of Ethiopia. Thus, besides taking the right measures by the concerned bodies to control further expansion of resistance against chloquine, more study on the degree of dissemination of the resistance pattern within the *P.vivax *strains in other malaria endemic regions of the country is recommended to have clear picture of the country wide distribution of the problem.

## Abbreviations

ACPR: Adequate Clinical and Parasitological Response; CQ: Chloroquine; CRPv: Chloroquine Resistant *Plasmodium vivax; *DACA: Drug Administration and Control Authority of Ethiopia; PRR: Parasite Reduction Ratio; SNNPR: South Nations and Nationalities Peoples Region; WBC: White Blood Cells; WHO: World Health Organization

## Competing interests

The authors declare that they have no competing interests.

## Authors' contributions

TK was fully involved in all phases of the study, including data collection and monitoring both in the field and laboratory, data analysis, interpretation, and write-up of the manuscript; KB was involved in data collection and field monitory, statistical analysis of data and critical revision of the manuscript for publication; KG sketched map of the study site and involved in data collection. All authors read and approved the final version of the manuscript.
